# Randomized phase III clinical trial comparing the combination of capecitabine and oxaliplatin (CAPOX) with the combination of 5-fluorouracil, leucovorin and oxaliplatin (modified FOLFOX6) as adjuvant therapy in patients with operated high-risk stage II or stage III colorectal cancer

**DOI:** 10.1186/s12885-015-1406-7

**Published:** 2015-05-10

**Authors:** Dimitrios Pectasides, Vasilios Karavasilis, George Papaxoinis, Georgia Gourgioti, Thomas Makatsoris, Georgia Raptou, Eleni Vrettou, Joseph Sgouros, Epaminontas Samantas, George Basdanis, Pavlos Papakostas, Dimitrios Bafaloukos, Vassiliki Kotoula, Haralambos P. Kalofonos, Chrisoula D. Scopa, George Pentheroudakis, George Fountzilas

**Affiliations:** 1 Oncology Section, Second Department of Internal Medicine, “Hippokration” Hospital, Athens, 11527 Greece; 2 Department of Medical Oncology, “Papageorgiou” Hospital, Aristotle University of Thessaloniki School of Medicine, Thessaloniki, Greece; 3 Section of Biostatistics, Hellenic Cooperative Oncology Group, Data Office, Athens, Greece; 4 Division of Oncology, Department of Medicine, University Hospital, University of Patras Medical School, Patras, Greece; 5 Department of Pathology, Aristotle University of Thessaloniki School of Medicine, Thessaloniki, Greece; 6 Third Department of Medical Oncology, “Agii Anargiri” Cancer Hospital, Athens, Greece; 7 First Propaedeutic Department of Surgery, “AHEPA” Hospital, Aristotle University of Thessaloniki School of Medicine, Thessaloniki, Greece; 8 Department of Medical Oncology, “Hippokration” Hospital, Athens, Greece; 9 First Department of Medical Oncology, “Metropolitan” Hospital, Piraeus, Greece; 10 Laboratory of Molecular Oncology, Hellenic Foundation for Cancer Research, Aristotle University of Thessaloniki School of Medicine, Thessaloniki, Greece; 11 Department of Pathology, University Hospital, University of Patras Medical School, Patras, Greece; 12 Department of Medical Oncology, Ioannina University Hospital, Ioannina, Greece

**Keywords:** Colorectal cancer, Adjuvant chemotherapy, Modified FOLFOX6, Capecitabine, Oxaliplatin, Prognosis, KRAS, Microsatellite instability

## Abstract

**Background:**

The aim of the trial was to compare two active adjuvant chemotherapy regimens in patients with early stage colorectal cancer (CRC).

**Methods:**

Patients were assigned to oxaliplatin, leucovorin and 5-FU for 12 cycles (group A, FOLFOX6) or oxaliplatin and capecitabine for eight cycles (group B, CAPOX). Primary endpoint was disease-free survival (DFS). Tumors were classified as mismatch repair proficient (pMMR) or deficient (dMMR) according to MLH1, PMS2, MSH2 and MSH6 protein expression. KRAS exon two and BRAF V600E mutational status were also assessed.

**Results:**

Between 2005 and 2008, 441 patients were enrolled, with 408 patients being eligible. After a median follow-up of 74.7 months, 3-year DFS was 79.8 % (95 % CI 76.5–83.4) in the FOLFOX group and 79.5 % (95 % CI 75.9–83.1) in the CAPOX group (p = 0.78). Three-year OS was 87.2 % (95 % CI 84.1-91.1) in the FOLFOX and 86.9 % (95 % CI 83.4–89.9) in the CAPOX group (p = 0.84). Among 306 available tumors, 11.0 % were dMMR, 34.0 % KRAS mutant and 4.9 % BRAF mutant. Multivariate analysis showed that primary site in the left colon, earlier TNM stage and the presence of anemia at diagnosis were associated with better DFS and overall survival (OS), while grade one–two tumors were associated with better OS. Finally, a statistically significant interaction was detected between the primary site and MMR status (p = 0.010), while KRAS mutated tumors were associated with shorter DFS. However, the sample was too small for safe conclusions.

**Conclusions:**

No significant differences were observed in the efficacy of FOLFOX versus CAPOX as adjuvant treatment in high-risk stage II or stage III CRC patients, but definitive conclusions cannot be drawn because of the small sample size.

**Trial registration:**

ANZCTR 12610000509066. Date of Registration: June 21, 2010.

**Electronic supplementary material:**

The online version of this article (doi:10.1186/s12885-015-1406-7) contains supplementary material, which is available to authorized users.

## Background

Colorectal cancer (CRC) is the third most common malignancy and the third leading cause of cancer death in the US [1]. Approximately half of the patients diagnosed with colorectal cancer will be cured after surgery and adjuvant treatment, while the rest will die from metastatic disease [2]. The role of adjuvant chemotherapy in stage III colon cancer is well established, according to several landmark randomized phase III trials. It has been shown that it reduces the risk of recurrence by 19–40 % and the risk of death by 16–33 % [3]. However, the role of adjuvant chemotherapy in patients with operated stage II colon cancer has not been clearly defined as yet. Current guidelines suggest the administration of adjuvant chemotherapy in stage II colon cancer patients when high-risk clinicopathological features are present [4].

The combination of oxaliplatin with leucovorin and bolus/infusional 5-fluorouracil (5-FU), called FOLFOX4, is the best-studied regimen in colorectal cancer and has proven its beneficial role as adjuvant chemotherapy [3, 5]. However, this regimen has in fact been replaced by modified FOLFOX6 (mFOLFOX6), which can be easily administered by a central venous catheter, avoiding hospitalization, and is accepted as equally effective as FOLFOX4 by the oncologic community. Capecitabine is an orally administered fluoropyrimidine prodrug that is biotransformed into active metabolites within cancer cells and mimics 5-FU infusion when it is administered twice daily. Capecitabine showed similar efficacy compared to bolus 5-FU/LV as adjuvant treatment in the X-ACT clinical trial [6], whereas its combination with oxaliplatin (CAPOX) demonstrated improved disease-free survival rate compared to bolus 5-FU/LV [7]. However, as far as we know, a formal comparison between CAPOX and FOLFOX in the adjuvant setting, in the context of a randomized trial, has never been performed.

The primary objective of the present prospective randomized clinical trial was to compare 3-year disease-free survival (DFS) rates between the two treatment schedules, mFOLFOX6 to the combination of oxaliplatin and capecitabine (CAPOX). Secondary endpoints were 3-year overall survival (OS) rates and the toxicity profile of therapies administered. An exploratory objective was the study of clinicopathological characteristics and biomarkers for potential prognostic and predictive utility.

## Methods

### Patients

In this multicenter prospective randomized phase III trial, patients with completely resected high-risk early stage CRC were enrolled. All patients had histologically confirmed high-risk American Joint Committee on Cancer (AJCC) stage II or stage III CRC. According to the protocol, high-risk features for stage II disease were high histological grade, lymphovascular or perineural invasion, mucinous component, T4 stage, extramural vein invasion, symptomatic bowel obstruction or perforation at diagnosis and less than 12 lymph nodes removed. Surgical resection with no residual disease should have been performed 4–8 weeks before enrollment, while adequate performance status (PS 0–1) and organ function had been confirmed. Exclusion criteria were the presence or history of malignant tumors, other than non-melanoma cancer of the skin and *in-situ* cervical cancer, severe cardiac disease, uncontrolled metabolic disorders or serious uncontrolled active infection, inflammatory bowel disease, loss of proximal gastrointestinal tract integrity, malabsorption syndrome, current history of chronic diarrhea, gastrointestinal hemorrhage or peptic ulcer, other serious concomitant diseases and a compromised general condition, including major neurological and psychiatric disorders, pregnancy or breastfeeding.

### Treatment

Patients were randomly assigned to receive oxaliplatin 85 mg/m^2^ on day 1, leucovorin 200 mg/m^2^ as a 2-h infusion on day 1 and 5-FU 400 mg/m^2^ IV bolus on day 1 followed by a 5-FU 2,400 mg/m^2^ 46-h continuous infusion, repeated every 14 days for 12 cycles (group A, modified [m]FOLFOX6) or oxaliplatin 130 mg/m^2^ on day 1 and capecitabine (Xeloda®) 1,000 mg/m^2^ bid on days 1–14, repeated every 21 days for eight cycles (group B, CAPOX). During randomization, done centrally at the Hellenic Cooperative Oncology Group (HeCOG) data office, patients were stratified according to the AJCC stage (high-risk II versus III). All patients with rectal primaries received adjuvant radiotherapy 46 Gy to the pelvic area and a 4 Gy boost, for a total dose of 50 Gy, concomitantly with capecitabine 825 mg/m^2^ twice daily on the days of radiotherapy, according to the existing local guidelines at that time. Two cycles of chemotherapy were administered before and the rest, 10 cycles for mFOLFOX6 or six cycles for CAPOX, after the completion of chemoradiotherapy.

All adverse events were recorded according to the National Cancer Institute Common Toxicity Criteria (NCI CTC) version 2.0 grading scale. The first cycle was administered according to the inclusion criteria described above. Subsequent cycles were not administered unless the granulocyte number was ≥1,500/mm^3^, platelet number ≥100,000/mm^3^ and all non-hematological toxicities resolved to grade ≤1. In case of a 2-week delay, treatment could be interrupted, according to the investigator’s decision. Capecitabine was interrupted in case of hand-foot syndrome, mucositis or diarrhea grade two, until these toxicities were resolved. The capecitabine doses that had been omitted were not given at a later point. Administration of G-CSF and recombinant erythropoietin was allowed. Also, oral pyridoxine was allowed, administered as prophylaxis or as the treatment of existing hand-foot syndrome. Finally, oxaliplatin was permanently interrupted in case of neurotoxicity grade ≥3.

### Evaluation of disease

Follow-up evaluation for disease recurrence was carried out after the completion of treatment in all patients, every 3 months for the first year, every 4 months for the second and third year and every 6 months for the fourth and fifth year, by serum carcinoembryonic antigen (CEA), chest X-rays and computed tomography (CT) scans of the abdomen and pelvis. Chest CT scans and MRI or bone scans were allowed when indicated.

The clinical protocol was approved by Institutional Review Boards (IRBs) in participating institutions (shown in Additional file 1: Table S1) and by the National Organization for Medicines. The trial was included in the Australian New Zealand Clinical Trials Registry on the 21^st^ of June 2010 and allocated the following Registration Number: ANZCTR 12610000509066. Written informed consent for participation in the trial was obtained from all the patients and optionally a separate informed consent was obtained for providing biological material for research purposes.

### KRAS and BRAF genotyping

A total of 328 formalin-fixed paraffin-embedded tumor tissue samples were processed for tissue microarray (TMA) construction with the Alpheys Minicore 3 Tissue Microarray system (Plaisir, France). For each case, three tumor and, where possible, three normal 1mm cores were embedded into the recipient block.

Upon histological evaluation, 319 tumors were available for DNA extraction from 8 um TMA core sections with >30 % tumor cell content. Following deparaffinization, the VERSANT Sample 1.0 Reagent Kit (Siemens Healthcare Diagnostics, Tarrytown, NY) was used for magnetic beads DNA isolation, according to the manufacturer’s instructions. Genotyping was performed with dd-sequencing on nested PCR products with intronic primers spanning the entire exons of interest, as follows: KRAS exon 2, 1^st^ PCR forward 5′-CGTCTGCAGTCAACTGGAATTT-3′ and reverse 5′-TTACTGGTGCAGGACCATTCTTT-3′; nested forward 5′-TTTAACCTTATGTGTGACATGTTCTAA-3′ and reverse 5′-GCATATTACTGGTGCAGGACCA-3′.

BRAF exon 15, 1^st^ PCR forward 5′-ATAATGCTTGCTCTGATAGG-3′ and reverse 5′-GTGAATACTGGGAACTATGAA-3′; nested forward 5′-CTACTGTTTTCCTTTACTTAC-3′ and reverse 5′-GGGAACTATGAAAATACTATA-3′.

Nested primers were 5′-end M13 coupled. Sense and antisense sequencing was performed using M13 forward and reverse primers in 10 ul reactions with the Big Dye Terminator kit v.1.1 (Applied Biosystems/Life Technologies, Paisley, UK). Products were visualized upon capillary electrophoresis in an ABI3130XL genetic analyzer, base called and further analyzed with the Sequencing Analysis version 5.2 software (Applied Biosystems). A total of 307 tumors were informative for KRAS exon 2 and BRAF exon 15 mutation status (96.2 %).

### Mismatch repair (MMR) protein immunohistochemistry (IHC)

IHC was carried out on 2 um thick TMA sections with the following antibodies and conditions: MLH1, clone ES05 (Monosan, Uden, Netherlands) at 1:80 dilution; MSH2, clone 25D12 (Novocastra/Leica Microsystems, Wetzlar, Germany) at 1:40 dilution; MSH6, clone EP49 (DAKO, Glostrup, Denmark) at 1:60 dilution; and, PMS2, clone M0R4G (Novocastra/Leica Microsystems) at 1:50 dilution. All tests were performed using a Bond Max™ autostainer (Leica Microsystems) with diaminobenzidine as chromogen for protein-antibody complex visualization. Stains were evaluated by two pathologists (G.R. and E.V.) for all tumor and normal cores, along with external controls for assessing method performance. Each core was evaluated for nuclear staining intensity and distribution of positive cells at 200× and 400× [8]. Tumors were scored for (a) the incidence of positive cells as 0 (<10 % positive), 1 (10–30 % positive), 2 (30–70 % positive) and 3 (>70 % positive); and (b) for staining intensity as 0 (negative), 1 (mild), 2 (intermediate) and 3 (strong), in comparison to internal controls (lymphocytes, normal epithelia) [9–11]. Scores for each core were recorded. For the purposes of the present study, tumors were classified as positive for incidence and intensity categories 1–3 (≥10 % positive nuclei with mild to strong intensity).

### Statistical analysis

The design was that of a superiority trial. A sample of 800 patients was required for the study, to ensure an 80 % power at the 5 % level of significance, for a two-sided test of the hypothesis that a difference of ±5 % in 3-year DFS rate exists from a baseline 3-year DFS rate of 78.2 %. Considering a 3 % withdrawal rate, 824 patients needed to enter the study. An interim analysis based on the O’Brien Fleming boundary values was planned when half (50 %) of the DFS events (166 relapses) had been reached. The study would be ended prematurely if either a significant difference was detected or the alternative hypothesis was rejected at the interim analysis.

Enrollment was closed prematurely at 441 patients because of slow accrual. At the time of the analysis, a futility test was retrospectively undertaken showing that, even if the study was to continue recruitment to reach the pre-specified number of 824 patients, the probability of reaching the primary endpoint, considering the 3-year DFS as it was defined by the study design, would not have been more than 0.1.

Continuous variables were presented as medians with the corresponding range and categorical variables as frequencies with the respective percentages. Chi-square or Fisher’s exact tests and the non-parametric Mann–Whitney test were used for comparing patient and tumor characteristics.

OS was measured from the date of randomization to the date of patient’s death or last contact, while DFS was measured from the date of randomization to documented first recurrence, death without prior documented recurrence or last contact, whichever occurred first. Surviving patients were censored at the date of last contact. Time to event distributions were estimated using Kaplan-Meier curves and compared using log-rank tests. For all univariate tests, significance level was set at α = 0.05. Cox proportional hazards models were used to assess the relationship of OS and DFS with various clinical and histological variables. Concerning multivariate analyses, significance threshold for keeping a variable in the final model was set at α = 0.15. The following standard parameters were included in the multivariate analyses: age, gender, primary site, stage and anemia. Treatment groups, as well as the examined markers, were included in the final model, in order to determine whether they added independent prognostic information to the model containing the significant clinicopathological parameters. No adjustments for multiple comparisons were done. Analyses of survival parameters and objective response rates were performed in all randomized patients (intention to treat, ITT population), while analyses of toxicity and therapy characteristics were performed only in patients who did receive treatment (treated patient population).

The SAS software was used for statistical analysis (SAS for Windows, version 9.3, SAS Institute Inc., Cary, NC).

## Results

**Fig. 1:**
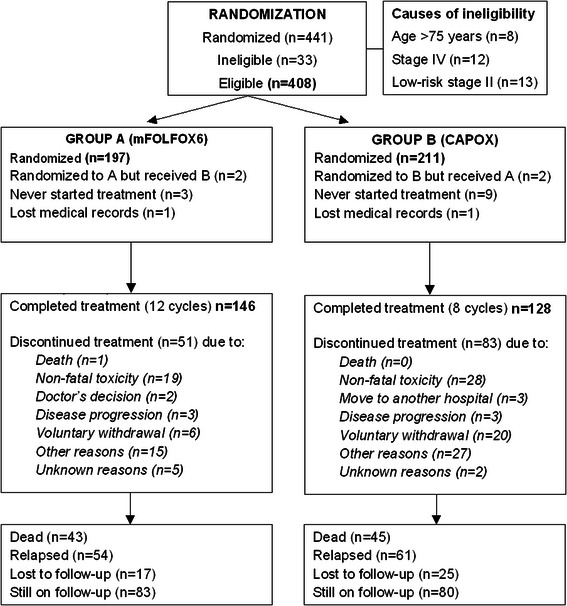
CONSORT diagram

Between November 2005 and January 2008, 441 patients were enrolled in the study. Among them, 408 patients (92.5 %) were eligible, with 197 randomized to group A (mFOLFOX6) and 211 to group B (CAPOX). The CONSORT diagram for the patient population is shown in Fig. 1. Table 1 shows the baseline characteristics of all eligible patients. The two groups of patients were balanced in selected patient and disease characteristics.

**Table 1:** Patient and disease characteristics

		Group A	Group B	*p*-value
		N = 197	N = 211	
Age	Median	62.4	63.7	0.45
	Range	23.7–74.7	36.6–75.0	
BMI	Median	26.75	26.90	0.89
	Range	17.9–34.6	17.3–35.0	
		N (%)	N (%)	
Gender	Female	86 (43.6)	94 (44.5)	0.91
	Male	111 (56.4)	117 (55.5)	
PS (ECOG)	0	177 (91.7)	195 (93.7)	0.37
	1	16 (8.3)	13 (6.3)	
	Unknown	4	3	
Anemia (Hb < 12g/dL)	No	148 (77.9)	161 (78.9)	0.75
	Yes	42 (22.1)	43 (21.1)	
	Unknown	7	7	
Weight loss	No	181 (94.8)	191 (93.6)	0.61
	Yes	10 (5.2)	13 (6.4)	
	Unknown	6	7	
TNM stage	II	61 (31.9)	68 (33.5)	0.66
	T3N0	54 (88.5)	60 (88.2)	
	T4N0	7 (11.5)	8 (11.8)	
	III	130 (68.1)	135 (66.5)	
	TxN1	81 (62.3)	83 (61.5)	
	TxN2	49 (37.7)	52 (38.5)	
	Unknown	6	8	
Histological grade	Grade 1	12 (6.3)	12 (5.9)	0.82
	Grade 2	139 (72.4)	152 (74.9)	
	Grade 3	41 (21.3)	39 (19.2)	
	Unknown	5	8	
Mucinous component	No	120 (67.8)	128 (72.7)	0.35
	Yes	57 (32.2)	48 (27.3)	
	Unknown	20	35	
Obstruction	No	167 (85.2)	179 (86.1)	0.84
	Yes	29 (14.8)	29 (13.9)	
	Unknown	1	3	
Perforation	No	181 (92.0)	190 (91.8)	0.95
	Yes	16 (8.0)	17 (8.2)	
	Unknown	0	4	
LVI/PNI (stage II)^a^	No	48 (80.0)	57 (85.1)	0.45
	Yes	12 (20.0)	10 (14.9)	
	Unknown	1	1	
Primary site	Right colon^b^	58 (29.5)	64 (30.8)	0.89
	Left colon^b^	86 (43.6 %)	87 (41.8 %)	
	Rectum	53 (26.9 %)	57 (27.4 %)	
	Unknown	0	3	

*BMI* body mass index, *PS* performance status, *Hb* hemoglobin; *Tx* any T

^a^Lymphovascular and/or perineural infiltration in patients with stage II disease

^b^Right colon: cecum, ascending colon, hepatic flexure, transverse colon; Left colon: splenic flexure, descending colon, sigmoid

### Treatment

In total, 196 patients received 2,022 cycles of mFOLFOX6 (median, 12; range, 1–12) and 207 patients received 1,372 cycles of CAPOX (median, 8; range, 1–8). Among them, 146 patients (74.1 %) completed treatment with mFOLFOX6 and 128 (61.0 %) with CAPOX. In total, in the mFOLFOX6 group (group A), 47 patients (23.4 %) and 51 (25.4 %) required a dose reduction of oxaliplatin and 5-FU, respectively. In the CAPOX group (group B), 8 patients (3.8 %) and 130 (61.0 %) required a dose reduction of oxaliplatin and capecitabine, respectively. Median relative dose intensities in the mFOLFOX6-treated patients were 98.8 % (range, 13–100 %) for oxaliplatin, 99.7 % (range, 42–100 %) for bolus 5-FU and 99.5 % (range, 43–100 %) for the continuous infusion 5-FU, while in the CAPOX-treated patients dose intensities were 99.3 % (range, 19–100 %) for oxaliplatin and 82.8 % (range, 29–100 %) for capecitabine.

### Toxicity

Adverse events of all grades, seen in ≥5 % of the patients, are shown in Table 2. The most common grade three–four toxicities were neutropenia (26.9 % of patients with mFOLFOX6 versus [vs] 8.1 % with CAPOX, p < 0.0002) and sensory neuropathy (7.1 % of patients with mFOLFOX6 vs 4.3 % with CAPOX, p = 0.21). Vomiting was more frequent in the CAPOX group (1.57 % vs 0 %, p = 0.012). Other severe toxicities with different incidences between the two arms were diarrhea (4.0 % of patients with mFOLFOX6 vs 7.1 % with CAPOX, p = 0.18) and fatigue (1.0 % of patients with mFOLFOX6 vs 2.4 % with CAPOX, p = 0.23).

**Table 2:** Toxicities by maximum NCI CTC version 2.0 grade for each treatment arm (N, number of patients)

		Group							
		mFOLFOX6				CAPOX			
		Grade				Grade			
		1	2	3	4	1	2	3	4
Hemoglobin	N	115	10	1	.	105	21	2	.
	%	58.4	5.1	0.5	.	49.7	9.9	0.9	.
Leucocytes	N	65	60	5	.	77	35	5	.
	%	33.0	30.4	2.5	.	36.5	16.6	2.4	.
Neutrophils	N	22	57	40	13	61	48	16	1
	%	11.2	28.9	20.3	6.6	28.9	22.7	7.6	0.5
Febrile neutropenia	N	1	.	.	.	.	.	2	.
	%	0.5	.	.	.	.	.	0.9	.
Platelets	N	75	26	5	.	70	32	7	.
	%	38.1	13.2	2.5		33.2	15.2	3.31	.
Nausea	N	34	9	2	.	36	13	3	.
	%	17.3	4.6	1.0		17.1	6.2	1.4	
Vomiting	N	17	14	.	.	19	13	5	1
	%	8.6	7.1			9.0	6.2	2.4	0.5
Mucositis	N	9	3	2	.	4	1	.	.
	%	4.6	1.5	1.0		1.9	0.5		
Diarrhea	N	26	12	6	2	27	28	12	3
	%	13.2	6.1	3.0	1.0	12.8	13.0	5.7	1.4
Constipation	N	19	4	.	.	15	5	.	.
	%	9.6	2.0			7.6	2.4		
Liver toxicity	N	71	17	2	.	69	7	.	.
	%	36.0	8.6	1.0		32.7	3.3		
Neuropathy	N	59	43	14	.	66	44	9	.
	%	29.9	21.8	7.1		31.2	20.8	4.3	
HandFoot	N	4	1	.	.	10	3	1	.
	%	2.1	0.5			4.7	1.4	0.5	
Alopecia	N	6	2	.	.	2	1	.	.
	%	3.0	1.0			0.9	0.5		
Allergic reaction	N	4	9	2	.	5	1	4	.
	%	2.0	4.6	1.0		2.4	0.5	1.9	
Fever	N	9	5	.	.	13	3	.	.
	%	4.6	2.5			6.2	1.4		
Infection	N	.	4	1	.	.	4	1	.
	%	.	2.0	0.5			1.9	0.5	
Fatigue	N	23	7	2	.	28	8	4	1
	%	11.6	3.6	1.0		13.2	3.8	1.9	0.5
Dizzines	N	6	1	.	.	8	.	.	.
	%	3.0	0.5			3.8			
Musculoskeletal pain	N	5	2	.	.	7	2	.	.
	%	2.5	1.0			3.3	0.9		
Metabolic	N	18	.	.	.	17	2	.	.
	%	9.1				8.1	0.9		

*NCI CTC* National Cancer Institute Common Toxicity Criteria

### Survival

**Fig. 2:**
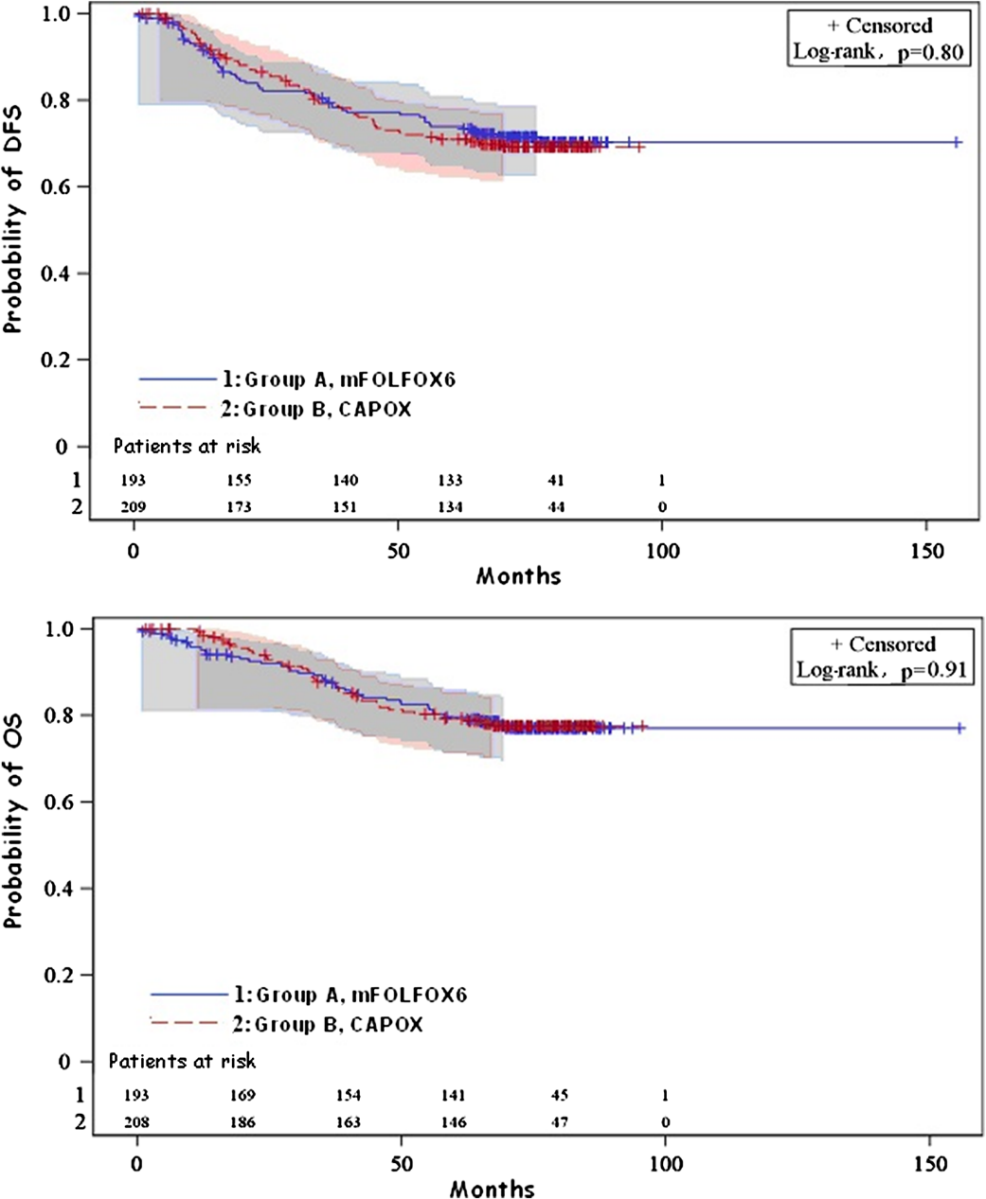
Survival curves for disease-free survival and overall survival in the two treatment arms. Shaded areas represent 95 % Hall-Wellner bands

After a median follow-up of 74.7 months (range 0–155.5 months), 54 patients (27.4 %) relapsed and 43 (21.8 %) died in the mFOLFOX6 group, while 61 patients (28.9 %) relapsed and 45 (21.3 %) died in the CAPOX group. Median DFS has not been reached for both groups, while 3-year DFS was 79.8 % (95 % confidence intervals [CI] 76.5–83.4) in the mFOLFOX6 group and 79.5 % (95 % CI 75.9–83.1) in the CAPOX group (p = 0.784). Median OS has also not been reached in both groups, while 3-year OS was 87.2 % (95 % CI 84.1–91.1) in the mFOLFOX6 group and 86.9 % (95 % CI 83.4–89.9) in the CAPOX group (p = 0.844). Kaplan-Meier curves for OS and DFS according to treatment are shown in Fig. 2. Overall, no significant survival differences were seen between the two treatment arms for either DFS or OS (mFOLFOX6 vs XELOX: Hazard ratio [HR] = 0.91, 95 % Confidence Intervals [CI] 0.58–1.44 and HR = 1.05, 95 % CI 0.68–1.60, respectively).

### MMR protein status and KRAS-BRAF mutations

Tumors were classified, according to IHC positivity for MLH1, PMS2, MSH2 and MSH6, in MMR proficient (pMMR) if all proteins were expressed and MMR deficient (dMMR) in case of null expression of at least one protein. Among 309 tumors informative for all MMR proteins, 34 (11.0 %) were dMMR and 275 (89.0 %) were pMMR. dMMR status was associated with absence of expression of MLH1 and/or PMS2 in 13 cases (38 %) and of MSH2 and/or MSH6 in 20 cases (59 %), while in one case only MSH2 was expressed. dMMR status was associated with higher grade and mucinous component in histology, while anemia was associated with earlier clinical stage (16.5 % for stage II versus 7.3 % for stage III, p = 0.014). Also, there was a trend for higher frequency of dMMR tumors in the right than in the left colon (p = 0.135) and in older patients (p = 0.08).

Among 307 tumors informative for *BRAF* and *KRAS* status, 15 (4.9 %) carried the V600E *BRAF* mutation, while 104 (34.0 %) were found to be *KRAS* mutants. Ninety-four tumors had codon 12 mutations (74 G12D, 7 G12C, 6 G12V, 4 G12S, 2 G12A and 1 G12R) and 10 had codon G13D mutations. No associations were noticed between mutations and basic patient or disease characteristics. *KRAS* and *BRAF* mutations were mutually exclusive. Also, *KRAS* or *BRAF* mutations were not associated with MMR status.

### Prognostic factor analysis

In univariate analysis, earlier AJCC clinical stage II versus III was associated with longer DFS (3-year DFS 92.0 % vs 73.8 %, respectively, HR = 0.31, 95 % CI 0.17–0.58, Wald’s p < 0.001) and OS (3-year OS 95.2 % vs 84.0 %, respectively, HR = 0.24, 95 % CI 0.12–0.47, p < 0.001), primary tumor location in the left colon with better OS (3-year OS 90.4 % vs 79.4 %, HR = 0.59, 95 % CI 0.38–0.91, p = 0.011) and anemia at the time of diagnosis with longer DFS (3-year DFS 85.2 % vs 78.1 %, HR = 0.29, 95 % CI 0.13–0.68, p = 0.008). Also, within stage III, patients with N2 disease had worse DFS (HR = 2.29, 95 % CI 1.53–3.44, p < 0.001) and OS (HR = 3.26, 95 % CI 2.01–5.29, p < 0.001) than those with N1. No conclusions could be made for T4N0 patients because their number was very low. Exploratory subgroup analysis showed that mutated *KRAS* was associated with shorter DFS in the subset of patients with left colon primary tumors (HR = 2.30, 95 % CI 1.17–4.52, p = 0.020) or stage II disease (HR = 1.88, 95 % CI 1.17–3.02, p = 0.010). In the group of rectal cancer patients, low rectal primary tumor location (first 5 cm from anal sphincter) was associated with worse DFS (3-year DFS 68.8 % vs 87.1 %, HR = 2.02, 95 %CI 1.27–5.31, p = 0.016) and OS (3-year OS 81.1 % vs 98.5 %, HR = 2.29, 95 % CI 1.19–5.31, p = 0.024) compared to middle (>5–10 cm) and upper (>10 cm) rectal tumors. Regarding the impact on DFS/OS, no significant interactions were observed between the treatment arm (FOLFOX or CAPOX) and any of the clinicopathological or molecular characteristics under study.

**Fig. 3:**
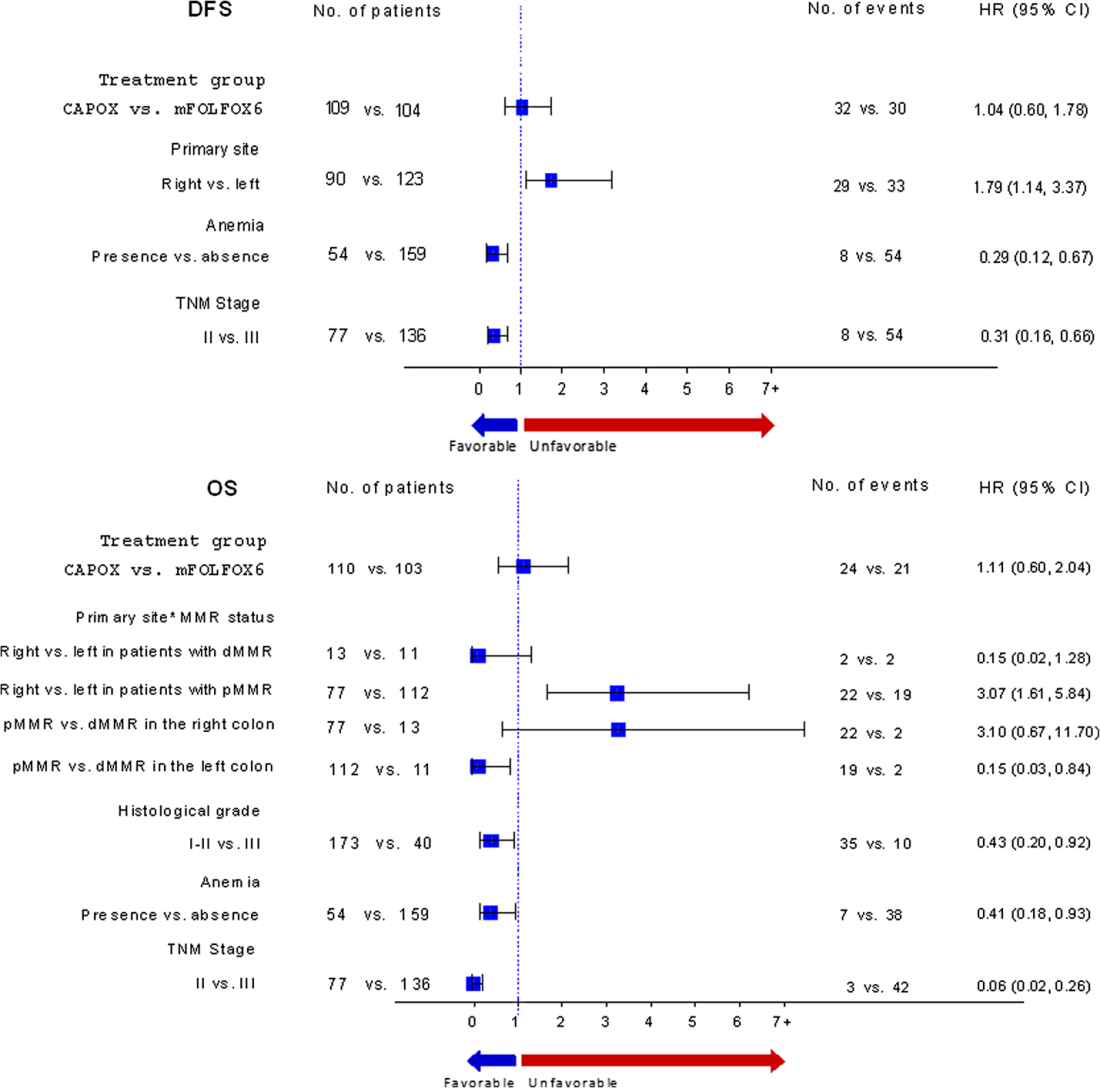
Forest plots demonstrating the multivariate analysis of prognostic factors for DFS and OS, including the interaction between MMR protein status and primary tumor location. MMR protein status significantly interacted with primary site only for OS and not for DFS. Position at the right side of the bar indicates adverse prognostic significance, whereas the opposite is consistent with favorable outcome. HR, hazard ratio; CI, confidence interval; DFS, disease-free survival; OS, overall survival; pMMR, proficient mismatch repair; dMMR, deficient mismatch repair

Multivariate analysis of prognostic factors showed that earlier TNM stage and the presence of anemia at diagnosis were associated with better DFS (HR = 0.31, 95 % CI 0.16–0.66, p = 0.002 and HR = 0.29, 95 % CI 0.12–0.67, p = 0.007, respectively) and OS (HR = 0.06, 95 % CI 0.02–0.26, p < 0.001 and HR = 0.41, 95 %CI 0.18–0.93, p = 0.035, respectively) (Fig. 3). Also, primary site location in the right colon was associated with worse DFS (HR = 1.79, 95 % CI 1.14–3.37, p = 0.010), while lower histological grade tumors were associated with better OS (HR = 0.43, 95 % CI 0.20–0.92, p = 0.031). Finally, dMMR was associated with shorter OS only in the left colon (p = 0.010 for interaction), as shown in Fig. 3, while mutated *KRAS* was associated with shorter DFS in the subset of patients with left colon tumors (HR = 2.30, 95 %CI 1.17–4.52, p = 0.020), but not in those with stage II disease.

## Discussion

In the present trial, patients with high-risk stage II or stage III surgically removed colorectal cancer were randomized between mFOLFOX6 and CAPOX, which are two established adjuvant chemotherapy regimens. FOLFOX has demonstrated its superiority over infusional 5-FU/LV in patients with stage III disease in the MOSAIC trial, with a 5-year DFS of 66.4 % and 6-year OS of 72.9 % [5]. Also, CAPOX was found superior to infusional 5-FU/LV in patients with stage III colon cancer with 3-year DFS reaching 70.9 % [7].

Our study demonstrated rather similar efficacy of the two regimens, with 3-year DFS and OS reaching 79.8 % and 87.2 %, respectively in the mFOLFOX6 group and 79.5 % and 86.9 %, respectively in the CAPOX group. Although the planned accrual was not achieved, retrospective futility analysis showed that superiority would have unlikely been shown in the case of completion. Also, this trial was not designed to demonstrate non-inferiority, while the inclusion of stage II patients might have weakened the sensitivity of the study to detect small differences, if any, in the efficacy of the two standard regimens. Nevertheless, this study contributes to the knowledge drawn from other trials that CAPOX is an acceptable regimen for the adjuvant treatment of colon cancer. One of them, the recently published AVANT trial [12], showed no difference between FOLFOX4 and CAPOX-bevacizumab in a much larger population, although it cannot be excluded that bevacizumab might have influenced the results. The only differences we could show between the two regimens were the necessity for placement of a central venous catheter for mFOLFOX6 and the distinct toxicity profile, with neutropenia appearing more frequently with mFOLFOX6 and vomiting with CAPOX. However, long-term toxicity, which is almost entirely represented by neuropathy, was nearly equally distributed between the two groups. Therefore, the choice between mFOLFOX6 and CAPOX should be discussed and guided according to doctors’ decisions and patients’ preferences.

The indication and choice of adjuvant chemotherapy in patients with stage II colon cancer have not been clarified as yet. Most trials showed no benefit in terms of DFS and OS and only the QUASAR study demonstrated a 29 % reduction in the risk for relapse at 2 years with adjuvant 5-FU/LV compared to observation [13]. Also, a recent meta-analysis showed that adjuvant chemotherapy offered a DFS and OS advantage in patients with stage II colon cancer, but the chemotherapy regimens were not all standard and the quality of the surgery was not always the best [14]. Therefore, current guidelines suggest adjuvant chemotherapy as an option in patients with stage II disease and high-risk features, such as grade 3 histology, vascular/lymphatic invasion, bowel obstruction or perforation, T4 primary with close, indeterminate or positive surgical margins, and low quality of surgery (less than 12 regional lymph nodes removed), according to a pooled analysis of seven randomized trials [15]. These data justify our decision to include patients with high-risk stage II colorectal cancer in our trial, though the regimen selection is still not adequately established. A post-hoc analysis of NSABP C-07 data [16], which was published after the completion of the present study, demonstrated that only DFS but not OS was improved by the addition of oxaliplatin to 5-FU/LV in patients with operated stage II colon cancer.

Recent progress in preclinical and translational research has shed light on multiple aspects of the molecular biology of colon cancer. Several lines of evidence confirmed the classification of colon cancer in two major groups; one characterized by chromosomal instability (CIN) and another harboring defects in mismatch repair enzymes (dMMR) leading to a hypermutated phenotype, otherwise called microsatellite instability (MSI). These defects are attributed either to germline mutations of the MMR enzymes (Lynch syndrome) or to acquired methylation of the promoter region of the *MLH1* gene [17]. The present study, in accordance to what has already been shown in the literature [18], showed that dMMR was more often associated with older age, earlier clinical stage and right-sided colon tumors of mucinous or high-grade histology. The role of *BRAF* V600E and *KRAS* mutations in the classification of early stage colon cancer is not well-defined. *BRAF* mutations were observed in only 15 (4.9 %) of the patients in our study, so no conclusions could have been made. In contrast, *KRAS* mutations were detected much more frequently, but no association with clinicopathological characteristics was found.

The above genetic alterations have been extensively examined by multiple groups for their possible prognostic significance. The role of MMR status is in a large part well-defined. It is generally accepted that dMMR confers favorable prognosis in patients with resected colon cancer [19–23] and thus patients with stage II dMMR tumors are usually not considered for adjuvant chemotherapy. However, the role of MMR status in the treatment of stage III disease remains controversial. Recently, two large relevant studies have been published [24, 25]. In the study of Sinicrope et al. [24], MMR status was not found to be prognostic in patients with resected stage III colon cancer, probably due to interaction with primary tumor site. Specifically, although dMMR conferred favorable prognosis in right-sided tumors, it had the opposite role in the left colon. These findings were confirmed by the present study, however the small sample size did not allow us to reach strong statistical significance. In contrast, very recently, Klingbiel et al. [25] showed that MSI high status is associated with prolonged relapse-free survival, irrespectively of the primary site. No doubt, large validation series are needed to obtain definitive answers to this question. The prognostic role of *KRAS* mutations in resectable colon cancer remains until now controversial [25–31]. Sinicrope et al. [24, 30] found that *KRAS* mutations were associated with adverse prognosis specifically in pMMR tumors, while Blons et al. [31] showed that *KRAS* mutations conferred shorter DFS in patients with left colon primaries, which seem to be consistent with our findings. An interesting finding was also that anemia at presentation conferred a better outcome. Although this was shown in the multivariate analysis for DFS and OS, a plausible explanation would be that other as yet unidentified confounding factors, associated with favorable tumor biology, might have been associated with anemia in our study population.

## Conclusions

The present randomized clinical trial showed that mFOLFOX6 and CAPOX are equally effective as adjuvant treatments in patients with resected high-risk stage II or III colorectal cancer and are equivalent therapeutic options, justified by the generated evidence. The distinct toxicity profiles, along with patient preferences and comorbidities, should guide the choice of therapy.

## Additional file

Additional file 1: Table S1. Institutional review boards (IRBs) or scientific committees (SCs) that have approved the study.

## Abbreviations

AJCC: American joint committee on cancer
CAPOX: Capecitabine, oxaliplatin
CEA: Carcinoembryonic antigen
CIN: Chromosomal instability
CRC: Colorectal cancer
CT: Computed tomography
DFS: Disease-free survival
dMMR: Deficient mismatch repair
5-FU: 5-fluorouracil
HeCOG: Hellenic cooperative oncology group
HR: Hazard ratio
IHC: Immunohistochemistry
IRB: Institutional review boards
mFOLFOX6: Modified folinic acid, 5-fluorouracil, oxaliplatin
MSI: Microsatellite instability
NCI: National cancer institute
OS: Overall survival
pMMR: Proficient mismatch repair
PS: Performance status
TMA: Tissue microarray
