# Supplementary material for: Randomized phase III clinical trial comparing the combination of capecitabine and oxaliplatin (CAPOX) with the combination of 5-fluorouracil, leucovorin and oxaliplatin (modified FOLFOX6) as adjuvant therapy in patients with operated high-risk stage II or stage III colorectal cancer
Source: BMC Cancer. 2015 May 10;15:384. doi: 10.1186/s12885-015-1406-7 (PMC4445286; doi:10.1186/s12885-015-1406-7)
Supplement: Additional file 1: Table S1. — Institutional review boards (IRBs) or scientific committees (SCs) that have approved the study. [file 12885_2015_1406_MOESM1_ESM.docx]

**Supplementary Table S1.** Institutional review boards (IRBs) or scientific committees (SCs) that have approved the study.

| IRB “Papageorgiou” Hospital, Thessaloniki, Greece |
| --- |
| IRB “Alexandra” Hospital, Athens, Greece |
| IRB “Sotiria” Hospital, Athens, Greece |
| IRB University Hospital of Patras, Patras, Greece |
| IRB Ioannina University Hospital, Ioannina, Greece |
| IRB “Agii Anargiri” Cancer Hospital, Athens, Greece |
| IRB “Attikon” University Hospital, Athens, Greece |
| SC “Henry Dunant” Hospital, Athens, Greece |
